# Bottom-up synthesis of ordered metal/oxide/metal nanodots on substrates for nanoscale resistive switching memory

**DOI:** 10.1038/srep25537

**Published:** 2016-05-09

**Authors:** Un-Bin Han, Jang-Sik Lee

**Affiliations:** 1 Department of Materials Science and Engineering, Pohang University of Science and Technology (POSTECH), Pohang 790-784, Korea

## Abstract

The bottom-up approach using self-assembled materials/processes is thought to be a promising solution for next-generation device fabrication, but it is often found to be not feasible for use in real device fabrication. Here, we report a feasible and versatile way to fabricate high-density, nanoscale memory devices by direct bottom-up filling of memory elements. An ordered array of metal/oxide/metal (copper/copper oxide/copper) nanodots was synthesized with a uniform size and thickness defined by self-organized nanotemplate mask by sequential electrochemical deposition (ECD) of each layer. The fabricated memory devices showed bipolar resistive switching behaviors confirmed by conductive atomic force microscopy. This study demonstrates that ECD with bottom-up growth has great potential to fabricate high-density nanoelectronic devices beyond the scaling limit of top-down device fabrication processes.

Resistive switching random-access memory (ReRAM) with metal-insulator-metal (MIM) structure has advantages of fast switching speed, low operation voltage, and good scalability, and has therefore been widely investigated for future memory applications^1^,^2^,^3^,^4^,^5^. Metal-oxide-based ReRAM is considered as one of the most promising candidates for next-generation high-density nonvolatile memory devices^6^,^7^,^8^,^9^,^10^. Resistive switching is a change in electrical resistance between high-resistance state (HRS) and low-resistance state (LRS), and the physical phenomenon of memory operation; therefore, stable and reliable resistance changes in metal oxide materials are very important in ReRAM device applications. Resistive switching behavior has been observed in various oxides, such as NiO^11^,^12^,^13^, CuO_x_^14^,^15^,^16^,^17^,^18^,^19^, HfO_2_^7^,^20^,^21^,^22^, and Ta_2_O_3_^23^,^24^,^25^. These oxides are reported to be good candidates for use as the resistive switching layer in ReRAM devices. Among the various metal oxides, CuO_x_ is an attractive switching material because it can be synthesized at low cost and is non-toxic. In addition, CuO_x_-based memory devices show good reliability. Consequently, many researchers have studied application of CuO_x_ as a candidate for use in next-generation memory devices^16^,^17^,^26^.

Next-generation memory device applications demand devices with very high density that exceeds the current lithographic length scale limit. The bottom-up approach using self-assembled nanostructures is a promising solution for scaling down the memory devices.

To fabricate nanoscale ReRAM devices, self-assembled nanoporous templates have been used^27^,^28^,^29^,^30^,^31^,^32^,^33^,^34^,^35^. Among the various nanoporous templates, anodized aluminum oxide (AAO) has advantages such as thermal/mechanical stability and ease of controlling a wide range of pore diameters, inter-pore distances, and depth of pores^36^,^37^,^38^,^39^,^40^. However, it is very difficult to grow nanoscale materials/structures inside the nanoporous templates directly since the pore size is typically less than 100 nm and the aspect ratio (pore depth-to-diameter ratio) is high. To grow the nanoscale materials inside the pores electrochemical deposition (ECD) is used. ECD into AAO templates is a very versatile and facile way to deposit nanoscale nanodots with controlled diameter and thickness. The ECD enables control of the composition of deposited films by adjusting the current density (*J*), pH, and temperature of the solution (*T*_sol_)^16^,^41^. Therefore, the ECD of metals, semiconductors, and insulators has been used in many industrial applications^41^,^42^,^43^,^44^.

In this study, we used self-assembled AAO nanotemplates as masks for fabrication of uniform nanoscale Cu/CuO_x_/Cu ReRAM devices. Using well-ordered AAO templates, ECD enables successful growth of Cu-based nanodots with high density on Au-coated Si substrates. The advantage of this process is growth of metal/oxide/metal layer by sequential deposition of each layer with solution processes. In addition, there is no need to use vacuum deposition/lithography systems and etching/annealing processes for fabrication of nanoscale memory devices. The surface morphologies of AAO templates and nanoscale ReRAM devices were investigated using field-emission scanning electron microscopy (FE-SEM). The microstructure and crystal structure of the devices were analyzed using high-resolution transmission electron microscopy (HR-TEM) and x-ray diffraction (XRD). The topography of the Cu/CuO_x_/Cu nanodot memory devices was investigated using atomic force microscopy (AFM) and the resistive switching behavior of the CuO_x_-based memory devices was directly examined using conductive AFM. This work demonstrates that ECD with bottom-up growth has great potential to fabricate high-density nanoelectronic devices beyond the scaling limit of top-down device fabrication processes.

## Results and Discussion

**Figure 1:**
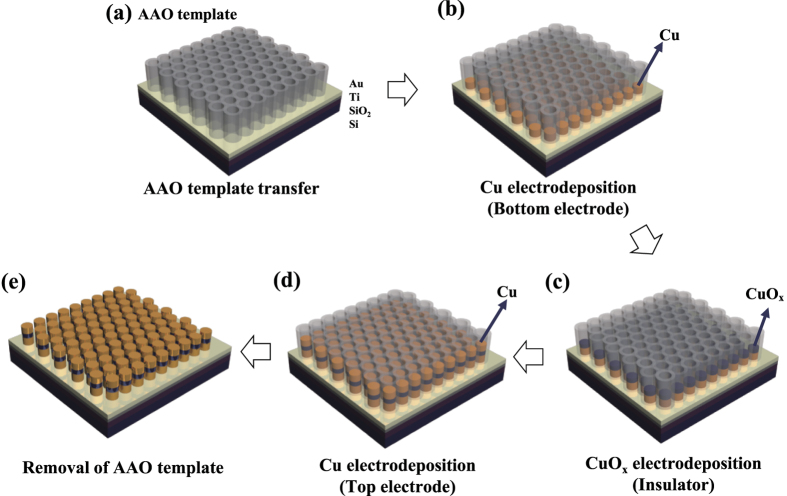
Schematic illustration of nanoscale resistive swtiching memory fabrication by bottom-up direct growth. (**a**) AAO nanotemplate transferred on conductive substrate. (**b**) Cu bottom electrode deposition by electrochemical deposition (ECD) through AAO nanotemplate mask. (**c**) CuO_x_ deposition by ECD. (**d**) Cu deposition by ECD. Cu/CuO_x_/Cu tri-layer was formed by bottom-up direct growth. (**e**) Final structure of nanoscale resistive switching memory composed of Cu/CuO_x_/Cu after removal of AAO template.

**Figure 2:**
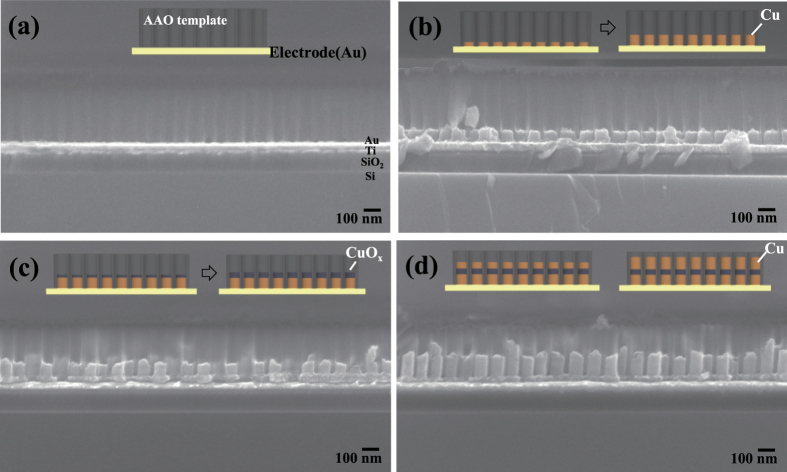
Bottom-up self-assembly and fabrication of nanoscale ReRAM devices by electrochemical deposition. Cross-sectional SEM images of Cu/CuO_x_/Cu nanodots electrodeposited on Au-coated Si substrate (Au/SiO_2_/Si). (**a**) AAO template transferred on the substrate. (**b**) Electrochemical deposition of Cu bottom electrode. (**c**) Electrochemical deposition of CuO_x_ on Cu/Au/SiO_2_/Si. (**d**) Electrochemical deposition of Cu on CuO_x_/Cu/Au/SiO_2_/Si.

Bottom-up self-assembly approaches were used to fabricate the nanoscale resistive switching memory devices. AAO was used as the template to obtain ordered nanoscale devices by the bottom-up approach^12^,^45^,^46^. The AAO is a self-assembled, nano-porous structure in which pore sizes, pore densities, and thicknesses can easily be controlled by adjusting the process parameters. In addition, pore diameters ranging from several-nanometers to several hundred-nanometers can be obtained. During growth of Cu and CuO_x_ onto the self-assembled AAO template, the metal ion reacts at surface of the electrode used for the substrate. Then the atoms are self-arranged from the bottom of the AAO template; they form a nanostructure from bottom-up self-assembly. Therefore, during the ECD of Cu, Cu nanodots can be formed by the reduction of Cu^2+^ ion in the solution starting from the bottom of AAO templates. Thicknesses can be controlled with the ECD time of Cu into the AAO template (Supporting Information, Fig. S1a–d). The thickness of Cu nanodots increased linearly with the deposition time because during ECD the amount of deposited material is proportional to the current, the deposition time, or both (Supporting Information, Fig. S1e). The Cu/CuO_x_/Cu nanodot arrays were fabricated as follows (Fig. 1). First, the AAO template with average pore diameter of 85 nm was transferred to the conductive substrates (Au/Ti/SiO_2_/Si). Cu bottom electrode/CuO_x_ resistive switching layer/Cu top electrode were sequentially deposited by ECD using AAO nanotemplates. After ECD, the AAO templates were removed by NaOH solution and finally highly-ordered, high-density nanoscale resistive switching memory devices were fabricated by bottom-up self-assembly. The AAO template was well placed on the Au-coated substrate (Fig. 2a), and the template pores were found to be well-aligned vertically. High-density arrays (Fig. 2b–d) of Cu/CuO_x_/Cu nanodots were obtained by ECD with an AAO template mask (~300 nm-thick, average pore diameter =  85 nm). The nanodots were well formed within the pores of the AAO template. There is some fluctuation in shapes and thickness of Cu/CuO_x_/Cu nanodots shown in Fig. 2(d). This may be due to the non-uniform cutting during the preparation of samples for cross-sectional SEM images.

**Figure 3:**
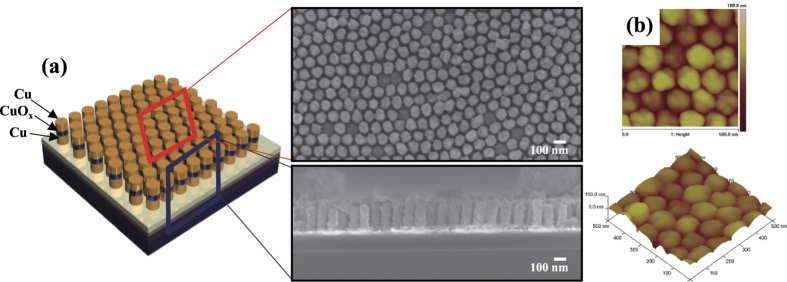
High-density nanoscale resistive switching memory devices by bottom-up direct growth. (**a**) Schematic illustration of the nanodot memory devices; plan and cross-sectional view SEM images of Cu/CuO_x_/Cu nanodots after removal of the AAO template. (**b**) Two and three-dimensional AFM images of Cu/CuO_x_/Cu nanodots.

**Figure 4:**
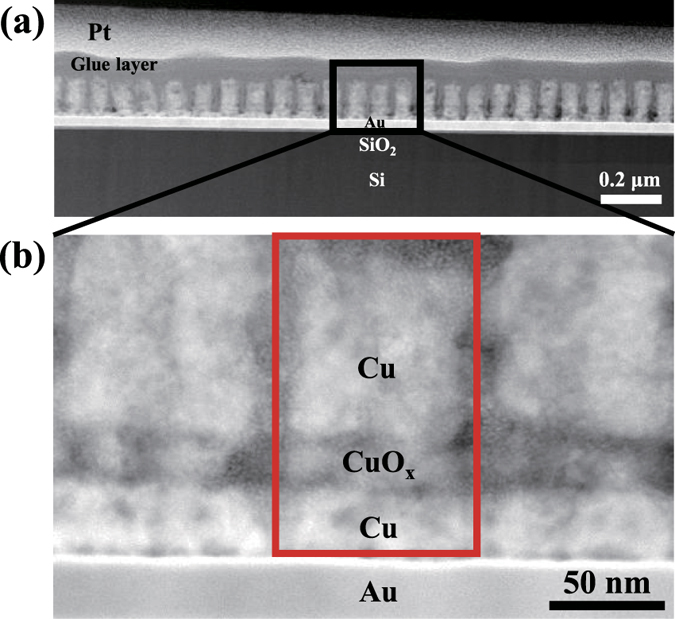
Microstructures of nanoscale ReRAM. (**a**) Cross-sectional high angle annular dark field (HAADF) TEM images of Cu/CuO_x_/Cu nanodots structure. (**b**) Detailed Cu/CuO_x_/Cu nanodots structure.

The final structure after removing the templates has almost uniform thickness shown in Figs 3 and 4. The total thickness of Cu/CuO_x_/Cu nanodots was ~180 nm. ECD provides the bottom-up growth of Cu/CuO_x_/Cu nanostructures from Au bottom electrodes to form ReRAM device structures as schematically shown in insets of Fig. 2. During ECD, the movement of the metal ions onto the AAO template was uniformly made by stirring the solution; this process increased metal ion mobility by eliminating air bubbles caused by the gas that originated from anions on the AAO template. Uniform deposition of nanodots is very important in this work, so solution was stirred at 200 rpm to prevent clogging of the AAO during metal deposition.

The formation of CuO_x_ and Cu nanodots in the AAO template can be explained by the charge transfer reactions and diffusion processes^47^,^48^,^49^. The formation of Cu and CuO_x_ is affected by pH, *J*, and *T*_sol_^49^,^50^,^51^. Temperature is one of the most important parameters to determine the overall reactions. In case of Cu, it can be deposited at high current density either at low or high temperatures. To form Cu metal as the bottom electrode, low temperature of 5 °C was selected since the deposition rate of Cu is very high at elevated temperatures. In case of CuO_x_, lower current density is required for proper synthesis of oxide layer. The deposition temperature (45 °C) is selected based on the growth rate of CuO_x_ layer.

In this study, CuO_x_ and Cu nanodots were deposited at a range of *J* at pH =  9. High *J* accelerated faster dissolution kinetics of Cu, so the concentration of Cu^2+^ ions increased and restricted the diffusion or migration of OH^–^ in the diffusion layer. Consequently, a metallic Cu layer is easily formed on the Au substrate:


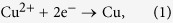


On the other hand, at low *J*, the OH^−^ ion shows higher diffusivity than that of Cu ions in bulk solution, resulting in easy combination of Cu^+^ with OH^−^ ions to form Cu_2_O:


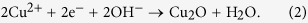


The surface morphologies of the Cu/CuO_x_/Cu nanodot memory devices (Fig. 3a) were investigated using FE-SEM. The SEM images of an array of Cu/CuO_x_/Cu nanodots were obtained after removal AAO nanotemplates. The bottom-up growth using the AAO nanotemplate mask produced uniform and well-ordered Cu/CuO_x_/Cu nanodots. Two- and three-dimensional AFM images (Fig. 3b) confirm that the array of Cu/CuO_x_/Cu nanodots was successfully synthesized with uniform dot size and thickness. Therefore, the bottom-up self-assembly processes using AAO templates successfully fabricated high-density nanoscale memory devices without using any lithography tools.

Further investigation on the morphology and crystal structure of the nanodots was conducted using cross-sectional TEM images of Cu/CuO_x_/Cu nanodot structure (Fig. 4a). Cu/CuO_x_/Cu tri-layer was confirmed from the magnified TEM image (Fig. 4b). The thickness of each layer is about 30 nm/20 nm/130 nm for Cu (bottom electrode)/CuO_x_/Cu (top electrode) measured using TEM image (Fig. 4b). Energy-dispersive X-ray spectroscopy (EDS) analysis was done to investigate the composition of each layer. It is clearly seen that Cu and CuO_x_ layer are distinguishable (Supporting Information Fig. S2). XRD results also revealed that Cu and CuO_x_ films could be well synthesized by controlling the current densities during ECD process (Supporting Information, Fig. S3). It is confirmed that Cu and CuO_x_ films fabricated by ECD were polycrystalline with (111) and (200) orientations due to the surface energy of face-centered cubic structure^19^. Cu film deposited on bare wafer with Au electrode showed reddish-brown, whereas CuO_x_ film showed dark blue (insets of Supporting Information, Fig. S3). In conjunction with TEM, XRD, and EDS analyses it is believed that Cu/CuO_x_/Cu structure was synthesized well by using bottom-up filling of each layer. It is very important to know the exact composition of each element in CuO_x_ resistive switching layer. Further analysis will be done to characterize the composition of copper and oxygen in CuO_x_ layer.

**Figure 5:**
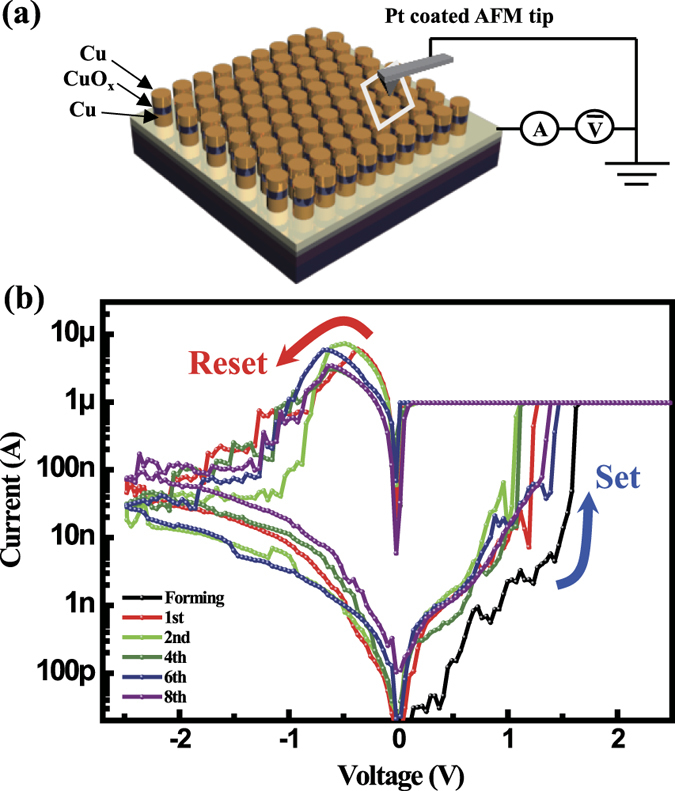
Schematic device measurement setup and programmable memory characteristics of nanoscale ReRAM. (**a**) Schematic measurement setup for electrical characterization of nanodot memory devices using conductive atomic force microscopy. (**b**) Resistive switching memory characteristics of nanoscale Cu/CuO_x_/Cu memory devices.

Electrical properties of nanoscale ReRAM devices were investigated by measuring current-voltage (I–V) characteristics of CuO_x_-based nanodots at room temperature using conductive AFM and a Pt-coated cantilever as a probe tip (Fig. 5a). The top electrode was grounded, and the electrical bias was applied to the bottom electrode. To achieve the first filament formation (e.g. forming operation), positive bias was applied to the bottom electrode up to 2.5 V in DC sweep mode. The current was abruptly jumped to the compliance current level at 1.5 V, as shown in Fig. 5b (black line). In this transition process, the resistance state of the device is changed from high resistance state (HRS) to the low resistance state (LRS) with a conductive filament formation. By contrast, the device is changed from LRS to HRS by filament rupture when the negative bias is applied to the bottom electrode. The fabricated devices showed typical bipolar resistive switching behavior (Fig. 5b). Thus we can program and erase the device by applying positive set bias and negative reset bias, respectively. The set operations occurred at about 1.3 V, and reset operations occurred at about − 0.75 V.

**Figure 6:**
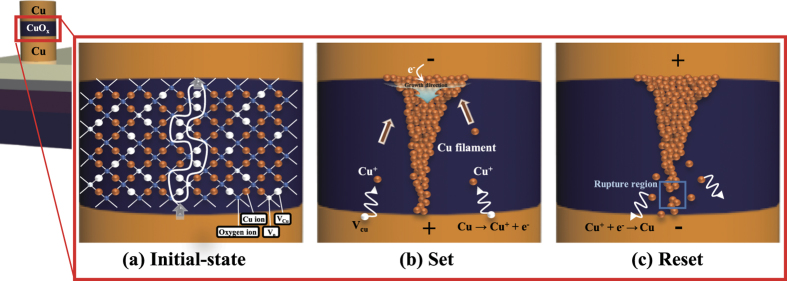
Resistive switching mechanism of nanoscale Cu/CuO_x_/Cu ReRAM. (**a**) Initial, (**b**) set, and (**c**) reset states.

To explain the resistive switching operations of the device (i.e. forming process, set, and reset), we considered Cu-ion migration in the device (Fig. 6a). The density and distribution of defects such as Cu^+^ ions in the switching layer are the important parameters for resistive switching operation. In Cu/CuO_x_/Cu nanodot memory devices, CuO_x_ used as the resistive switching layer has the high binding energy between Cu and oxygen ions, while Cu used as the electrode has a low ionization energy^23^,^52^. Thus metallic Cu in the bottom electrode is more easliy ionized to Cu^+^ ions and electrons (e^−^) than in the CuO_x_ switching layer when the positive bias is applied to the bottom electrode. Cu ions have high diffusivity and solubility in the CuO_x_. They are diffused from the bottom Cu electrode through the Cu vacancies of the CuO_x_ switching layer, and they are accumulated from the top electrode to the bottom electrode with electrons injected from the top electrode. That is, the Cu ion-based filament can grow from the top to the bottom in the resistive switching layer during applying a positive bias. This soft-breakdown of the device is called forming operation which is the first set operation (Fig. 6b). The operation polarity of the device is dependent on the initial forming operation. Strong one-directional electric field is applied to a device during the forming operation. This process induces asymmetric Cu^+^ ion distribution in the switching layer between the bottom and top electrodes because migration direction of Cu^+^ ions is determined by the initial forming bias polarity, resulting in an asymmetric electrical property of the device. Once the filament is formed in the switching layer by forming process, the conductive filament gradually dissolves during applying the opposite bias (Fig. 6c). This filament dissolution occur at the bottom region which has a relatively weak filament than that of the top region. Consequently, the repeated set and reset operations occur by filament formation and rupture at weak filament region. The conductive filament can be easily formed and ruptured by electrical bias due to Cu^+^ ion migration in the switching layer.

On/off ratio of the resistance states directly affects the sensing margin of memory devices. High on/off ratio is required for multilevel data storage and reliable reading operation. Our device shows on/off ratio of ~10^3^. Therefore, it is thought that the memory device fabricated by bottom-up processes can be used as the nonvolatile memory element in high-density memory applications.

To check the validity of using the AFM probe as the conductive probe tip, we measured the current of samples attached directly to the bottom electrode of the AFM probe (short circuit) and of samples that did not contact the top electrode (open circuit) (Supporting Information, Fig. S4). The current increased immediately to the compliance level (1 μ A); this result confirms that the AFM probe operated well as the conductive probe. Moreover, in the open circuit the current was very low (~picoamperes). These measurements confirm that the resistive switching phenomenon originated from the fabricated nanoscale memory devices.

In this work, the individual memory device was measured by using the conductive AFM. We can program/erase/read the individual memory elements by proper location of AFM tip since every device is separated by each other. In this study the main purpose is to demonstrate the possibility of fabrication of resistive switching memory devices *in situ* by ECD. The resistive switching properties are related to dimensions of nanodots. The layer thickness is related to set/reset voltages and the size of devices can determine the reset current. The device size is dependent on the pore diameter of templates and it is possible to change the diameter of templates by changing the AAO synthesis method. In addition, the oxide layer thickness can be controlled by deposition time. Comparative study is being done to change the dimensions of nanodevices by changing the pore diameters of AAO templates and by controlling the ECD processes.

In conclusion, we fabricated copper oxide-based ReRAM device using AAO as the template layer. Nanoscale memory devices were fabricated using bottom-up direct growth. ECD was used to synthesize Cu/CuO_x_/Cu nanodots on self-assembled nanoporous AAO templates. The ordered array of MIM-structured memory devices (Cu/CuO_x_/Cu) was successfully synthesized with uniform dot size and thickness. The fabricated memory devices showed reliable and reproducible resistive switching memory characteristics with the application of electrical biases. This method overcomes the scaling limits of currently-used nano device-fabrication methods.

## Methods

### Fabrication of self-assembled nano-templates

To fabricate nanoporous AAO templates, aluminum (Al) foil (99.999% purity, 0.50-mm thickness, Goodfellow) was used. AAO nanotemplates were fabricated using a two-step anodization process after electro-polishing to flatten the surface as described previously^40^. The first and second anodizations were performed in 0.3 M oxalic acid with a carbon cathode at 7 °C and 40 V for 24 h. After the second anodization, the widening process was performed in a 0.1 M H_3_PO_4_ solution at 30 °C. To remove the remaining Al layer, the AAO pores were filled with polystyrene (1.7 wt % PS/CHCl_3_ solution), then the substrate was washed in a saturated solution of HgCl_2_ with deionized water to separate the Al layer from AAO. The AAO/Polystyrene (PS) film was immersed in 0.1 M H_3_PO_4_ solution at 30 °C for 30 min to remove the barrier layer from the oxide/metal interface, then was transferred onto the substrate. Finally, the PS film on the AAO templates was removed by immersing it in CHCl_3_.

### Fabrication of nano-scale resistive switching memory devices

The Si substrate with a 100-nm SiO_2_ layer was used as the substrate for device fabrication. A 20-nm-thick Ti adhesion layer and a 50-nm-thick Au layer were deposited on the SiO_2_ layer by E-beam evaporation. The AAO template with 300-nm thickness was carefully transferred to the Au-coated Si substrate and dried at 80 °C for 20 min. Au is used as the seed layer for subsequent ECD. In addition, Au is used as the bottom contact for electrical measurement. Many kinds of metals can be used as the seed layer and the bottom contact, so other metals can be used for this purpose. ECD was used to deposit the Cu/CuO_x_/Cu nanodots sequentially on the substrate with the AAO template as the mask. The nanodots were synthesized from 0.6 M CuSO_4_·5H_2_O aqueous solution amended with 3 M lactic acid (Sigma Aldrich) to stabilize Cu (II) ions. The aqueous solution was adjusted to a pH of 9 by adding 2 M NaOH (Sigma Aldrich) then stirred overnight using a magnetic stirrer^53^,^54^. ECD of the nanodots into the AAO template was conducted in a two-electrode system using a carbon counter-electrode. ECD was performed using a DC power supply. ECD exploits electrically-driven redox reactions in the solution. On the basis of this mechanism, Cu nanodots as the bottom and top electrodes were deposited at *J* =  5 mA/cm^2^ at 5 °C for 30 s, then CuO_x_ nanodots as the resistive switching layer were deposited with *J* =  1 mA/cm^2^ at 45 °C for 30 s. By this processes Cu and CuO_x_ can be deposited by bottom-up growth on the substrates with AAO as the template. After deposition of Cu/CuO_x_/Cu nanodots, the substrate was immersed in 1 M NaOH solution for 30 min to remove the AAO template, then rinsed with deionized water to remove the remaining NaOH^40^.

### Characterization

The morphologies of the Cu/CuO_x_/Cu nanodots were observed using a field emission scanning electron microscope (FE-SEM; JSM 7401F, JEOL). The microstructure and crystal structure of nanodots were investigated using a high-resolution transmission electron microscope (HR-(S)TEM-I; JEM 2100F with a Cs corrector on STEM, JEOL) and by x-ray diffraction (XRD, D/MAX-2500/PC, RIGAKU) using Cu Kα radiation (λ  =  1.54178 Å). Before the TEM investigations, the samples were prepared using a focused ion beam (FIB; Helios, FEI).The surface morphologies of Cu/CuO_x_/Cu were examined using AFM (Dimension 3100 +  Nanoscope V, VEECO) in non-contact mode and the electrical properties were measured using conductive AFM (XE-100, Park systems) in contact mode; the scan rate was 1 Hz, the scan configuration was 256 ×  256 pixels, and the scan size was 500 nm ×  500 nm. The AFM measurements were performed at room temperature and atmospheric pressure. Micro- cantilevers (length : 225 μ m, Park Systems) with frequency of 75 kHz, a spring constant of ~2.8 N m^−1^ and a radius of curvature of ~25 nm were used.

## Additional Information

**How to cite this article**: Han, U.-B. and Lee, J.-S. Bottom-up synthesis of ordered metal/oxide/metal nanodots on substrates for nanoscale resistive switching memory. *Sci. Rep.* **6**, 25537; doi: 10.1038/srep25537 (2016).

## Supplementary Material

Supplementary Information
