# Supplementary material for: Bottom-up synthesis of ordered metal/oxide/metal nanodots on substrates for nanoscale resistive switching memory
Source: Sci Rep. 2016 May 9;6:25537. doi: 10.1038/srep25537 (PMC4860564; doi:10.1038/srep25537)
Supplement: Supplementary Information [file srep25537-s1.pdf]

Supplementary Information

Bottom-up synthesis of ordered metal/oxide/metal nanodots on substrates for nanoscale resistive switching memory

Un-Bin Han and Jang-Sik Lee*

Department of Materials Science and Engineering, Pohang University of Science and Technology

(POSTECH), Pohang 790-784, Republic of Korea

*E-mail: jangsik@postech.ac.kr

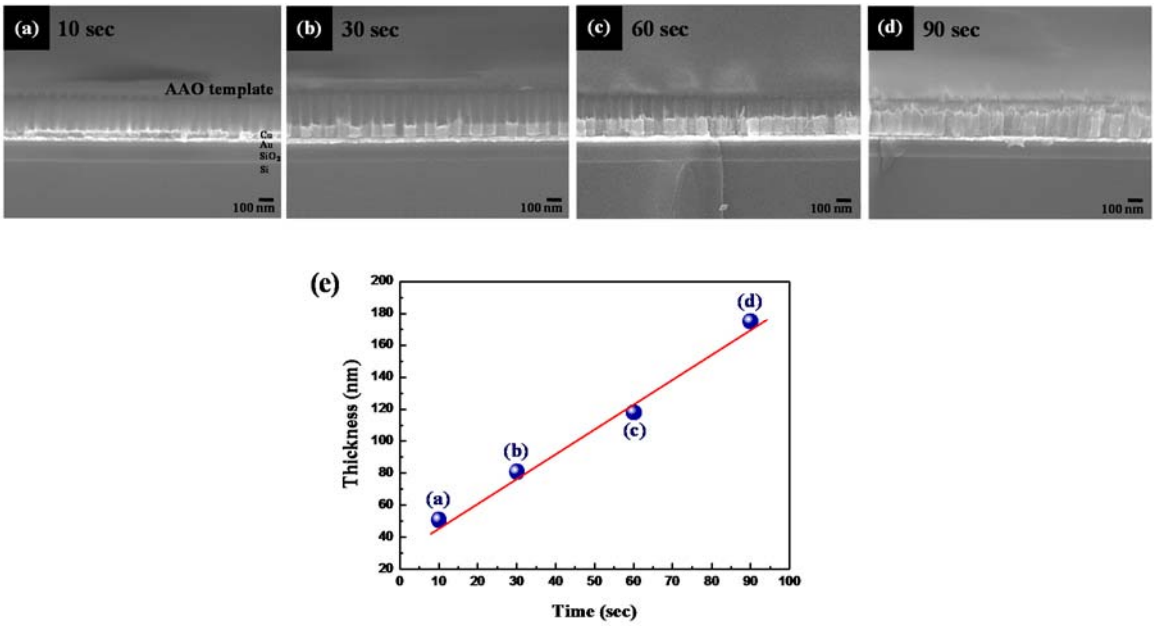

Figure S1. Cross-sectional SEM images of electrodeposited Cu obtained at different deposition time: (a) 10 sec. (b) 30 sec. (c) 60 sec. (d) 90 sec. (e) Plot of the deposition time versus the thickness obtained from Supplementary Figures S1(a), (b), (c), and (d).

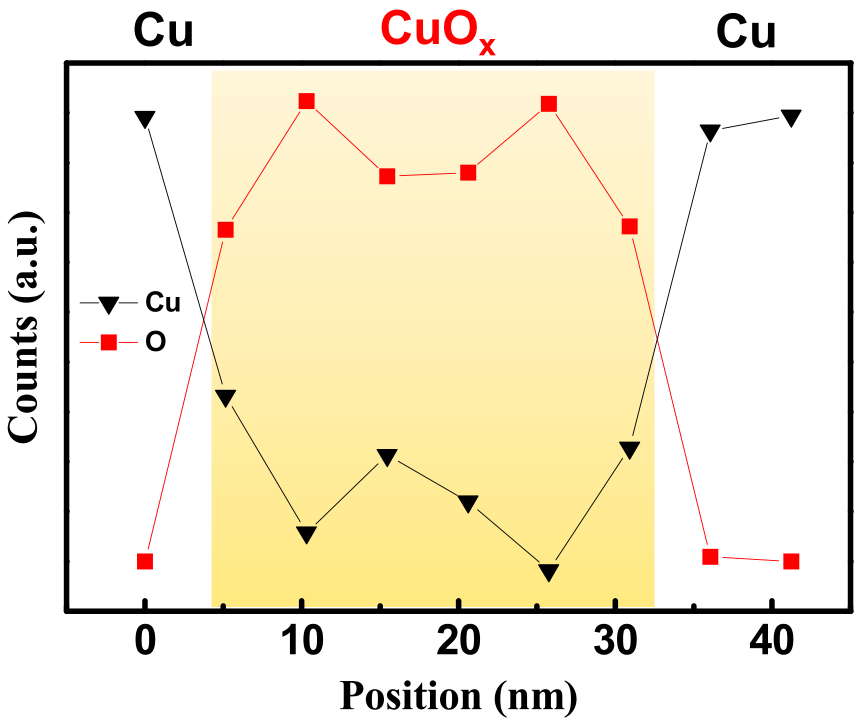

Figure S2. EDS line profile of Cu/CuO_x/Cu structure.

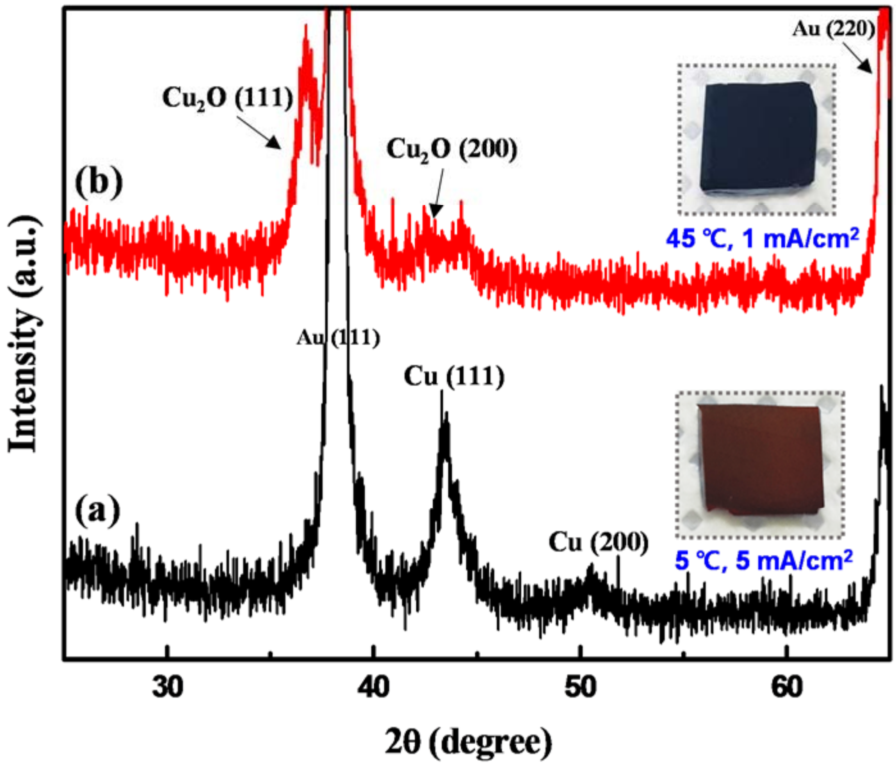

Figure S3. XRD patterns of Cu and CuO_x thin films obtained at different electrochemical deposition conditions: (a) current density of 5 mA/cm^2 at 5 ºC. (b) current density of 1 mA/cm^2 at 45 ºC.

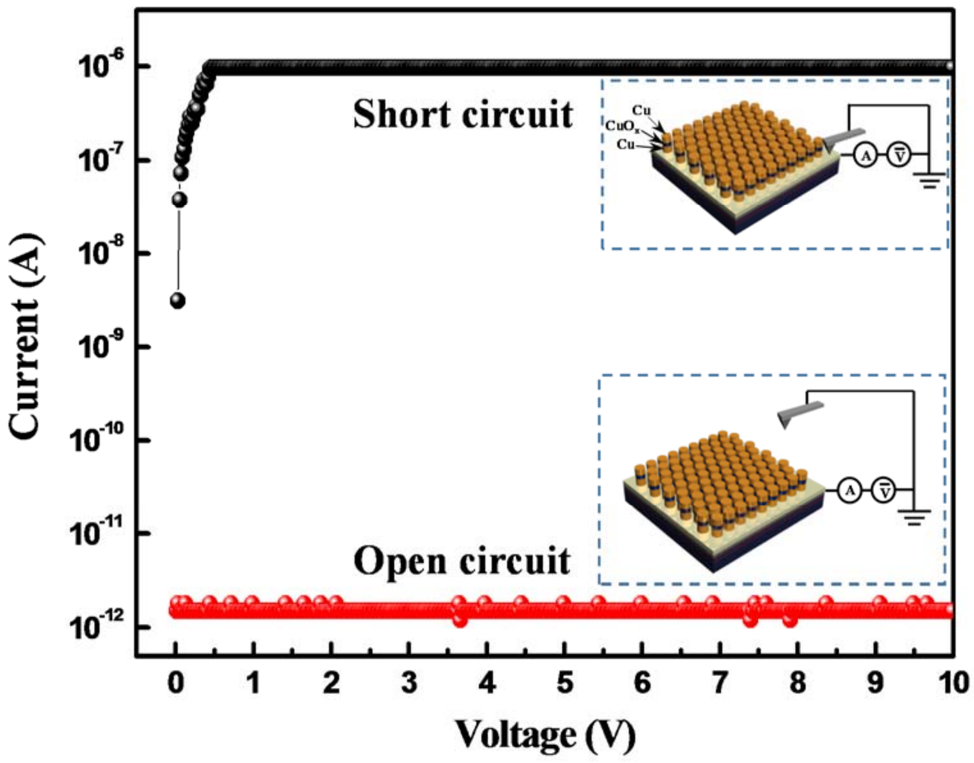

Figure S4. To determine the I-V responses of the nanodot memory device C-AFM was used. In the case of short circuit, the AFM probe was directly attached to the bottom electrode. In the case of open circuit, the probe did not contact the top electrode.
